# Hybrid neural network approaches to predict drug–target binding affinity for drug repurposing: screening for potential leads for Alzheimer’s disease

**DOI:** 10.3389/fmolb.2023.1227371

**Published:** 2023-06-27

**Authors:** Xialin Wu, Zhuojian Li, Guanxing Chen, Yiyang Yin, Calvin Yu-Chian Chen

**Affiliations:** ^1^ School of Computer Science and Technology, Guangdong University of Technology, Guangzhou, China; ^2^ Guangzhou University of Chinese Medicine, Guangzhou, China; ^3^ Artificial Intelligence Medical Research Center, School of Intelligent Systems Engineering, Shenzhen Campus of Sun Yat-Sen University, Shenzhen, China; ^4^ Department of Medical Research, China Medical University Hospital, Taichung, Taiwan; ^5^ Department of Bioinformatics and Medical Engineering, Asia University, Taichung, Taiwan

**Keywords:** Alzheimer’s disease, drug repurposing, hybrid neural network, molecular docking, sigma-1 receptor

## Abstract

Alzheimer’s disease (AD) is a neurodegenerative disease that primarily affects elderly individuals. Recent studies have found that sigma-1 receptor (S1R) agonists can maintain endoplasmic reticulum stress homeostasis, reduce neuronal apoptosis, and enhance mitochondrial function and autophagy, making S1R a target for AD therapy. Traditional experimental methods are costly and inefficient, and rapid and accurate prediction methods need to be developed, while drug repurposing provides new ways and options for AD treatment. In this paper, we propose HNNDTA, a hybrid neural network for drug–target affinity (DTA) prediction, to facilitate drug repurposing for AD treatment. The study combines protein–protein interaction (PPI) network analysis, the HNNDTA model, and molecular docking to identify potential leads for AD. The HNNDTA model was constructed using 13 drug encoding networks and 9 target encoding networks with 2506 FDA-approved drugs as the candidate drug library for S1R and related proteins. Seven potential drugs were identified using network pharmacology and DTA prediction results of the HNNDTA model. Molecular docking simulations were further performed using the AutoDock Vina tool to screen haloperidol and bromperidol as lead compounds for AD treatment. Absorption, distribution, metabolism, excretion, and toxicity (ADMET) evaluation results indicated that both compounds had good pharmacokinetic properties and were virtually non-toxic. The study proposes a new approach to computer-aided drug design that is faster and more economical, and can improve hit rates for new drug compounds. The results of this study provide new lead compounds for AD treatment, which may be effective due to their multi-target action. HNNDTA is freely available at https://github.com/lizhj39/HNNDTA.

## 1 Introduction

Alzheimer’s disease (AD) is a neurodegenerative disease that mainly affects elderly people and whose etiology remains unclear. The symptoms of patients include a decline in cognitive abilities and a weakening of memory and thinking abilities (Hung and Fu, 2017; Srivastava et al., 2021; Briggs et al., 2016). Although there are some drugs currently used to treat AD, their effectiveness is limited. However, drug repurposing (DR) has provided a new approach and selection for the treatment of AD (Padhi and Govindaraju, 2022; Ihara and Saito, 2020). This method involves reanalyzing the biological effects of known drugs and applying them to new areas of disease treatment. DR can accelerate the development of new drugs, provide more treatment options, and reduce the risk of drug development.

Previous studies have suggested that sigma-1 receptor (S1R) has neuroprotective effects and that its physiological function has a direct impact on endogenous neuroprotective mechanisms (Voronin et al., 2023). As a protein chaperone, S1R locates on specialized lipid rafts of mitochondria-associated endoplasmic reticulum membranes (MAMs), which are known to form mitochondrial endoplasmic reticulum contacts (MERCs) with the outer mitochondrial membrane and play a role in various biochemical processes, such as autophagosome formation, cellular energy production, and maintenance of *IR*3*R*3-dependent calcium homeostasis. Thus, disruption of this structure is now considered an early stage in the pathogenesis of neurodegenerative diseases, including AD. Activation of S1R using agonists has been shown to maintain the structural and functional stability of MAMs and MERCs, thereby enhancing autophagic activity, restoring mitochondrial function, and regulating intracellular calcium balance (Barazzuol et al., 2021; Leal and Martins, 2021; Wilson and Metzakopian, 2021; Weng et al., 2017). In AD models, such as PS1-KI and APP-KI, dendritic spines of hippocampal neurons are lost both *in vitro* and *in vivo*, indicating that the loss of mushroom-shaped “memory spines” reflects cognitive decline, learning, and memory deficits in AD (Ryskamp et al., 2019; Fisher et al., 2015), suggesting the involvement of reduced S1R in AD pathology. The mixed muscarinic/S1R agonist AF710B stabilizes mature mushroom spines in hippocampal cultures derived from AD mice *in vitro*, while pridopidine, an S1R agonist, stabilizes mushroom spines in an Alzheimer’s mouse model through its action on S1R. S1R agonists have demonstrated preclinical efficacy in AD animal models (Ryskamp et al., 2019; Fisher et al., 2015). Donepezil, a potent acetylcholinesterase inhibitor used for AD treatment, is also a high-affinity S1R ligand. Precise pharmacological studies on the interaction between donepezil and S1R suggest that the drug exerts anti-amnesic effects primarily through S1R activation against scopolamine, *β*-amyloid, or carbon monoxide-induced memory impairments (Hassan et al., 2017). Overall, S1R agonists exhibit neuroprotective effects and modulate synaptic plasticity, making S1R a potential target for AD treatment.

In the past decade, the “one disease–one target–one drug” paradigm has dominated the approach to drug discovery. However, this paradigm has certain limitations, and recent advances in systems biology have shifted the focus from “single-target drugs to “multi-target drugs” (Noor et al., 2023). When treating a particular disease, it is not feasible to rely solely on a single target to identify drugs. Instead, a range of targets within an imbalanced pathway in the complex biological network must be considered as inhibiting a single enzyme alone may lead to cancer cells compensating by activating other enzymes (Ryskamp et al., 2019; Fisher et al., 2015; Hassan et al., 2017). Zhi et al. utilized network pharmacology and molecular docking to reveal dihydroorotate dehydrogenase (DHODH) as a therapeutic target for small-cell lung cancer. Subsequently, they constructed a prediction model using graph neural networks (GNNs) and traditional machine learning methods to screen for potential DHODH inhibitors (Noor et al., 2023; Zhi et al., 2021). Cantini et al. introduced a multi-network strategy by integrating multiple genomic information layers, particularly gene co-expression and protein–protein interactions, to identify cancer-related targets. They employed consensus clustering algorithms in a predictive network, revealing CD46, BTG2, ATF3, HDGF, and F11R as driver genes in cancer (Noor et al., 2023; Cantini et al., 2015).

In drug repurposing, artificial intelligence (AI) plays an important role. By analyzing data on existing drugs and diseases using machine learning and deep learning methods, potential drugs can be quickly and efficiently screened (Cheng and Cummings, 2022; Yin and Wong, 2021; Vatansever et al., 2021). In addition, simulating the interactions between drugs and proteins can predict drug activity and affinity, guiding drug repositioning research. In recent years, researchers have successfully screened many promising drugs using AI methods (Selvaraj et al., 2021; Malandraki-Miller and Riley, 2021; Patel et al., 2020). These studies indicate that drug repositioning has important clinical application prospects, and AI methods can provide more powerful support for drug repositioning.

The affinity between drugs and targets is the basis for drug action, and predicting the affinity between drugs and targets is an important part of drug repurposing (Pushpakom et al., 2019; Parisi et al., 2020). Traditional experimental methods have disadvantages such as high cost and low efficiency, making it necessary to develop a fast and accurate prediction method. In recent years, with the development of deep learning technology, using neural networks to predict the affinity between drugs and targets has gradually become a research hotspot (Thomas et al., 2022; Choudhury et al., 2022; Jiang et al., 2022; Wang and Dokholyan, 2022). Neural networks are powerful computational tools with the ability to deal with non-linear problems and have achieved some success in predicting the affinity between drugs and targets.

In recent years, more and more researchers have begun to explore the use of neural networks to construct computational models for drug repositioning prediction to screen drugs for treating AD (Chyr et al., 2022; Wu et al., 2022; Siavelis et al., 2016). Some related studies have made some progress. For example, Zhou et al. Fang et al. (2022) proposed an integrated network-based AI method that can quickly translate genome-wide association study findings and multi-omics data into genotype-based therapeutic discoveries in AD, and identified pioglitazone as a potential new method for treating AD using AI methods. Tsuji et al. (2021) developed a deep learning-based computational framework that can extract low-dimensional representations of high-dimensional protein–protein interaction network data and infer potential drug target genes using latent features and state-of-the-art machine learning techniques. The study inferred that tamoxifen, bosutinib, and dasatinib could serve as repositionable candidate compounds against the disease. Rodriguez et al. (2021) proposed a machine learning framework, DRIAD (drug repositioning in AD), which quantifies potential associations between the pathological severity of AD and molecular mechanisms encoded in a list of gene names, and identified a ranked list of repositioning candidates for treating AD from 80 FDA-approved and clinically tested drugs.

Using AI methods for drug repurposing has become an important approach in AD drug research, providing important ideas and directions for new drug discovery. Although many studies have used neural networks to predict drug–target affinity, their application in the field of AD treatment is still relatively limited. This study aims to use neural networks to predict drug–target affinity and screen potential drugs for the treatment of AD, providing new ideas and choices for AD treatment. At the same time, we will compare and analyze different neural network models to find the best prediction model.

**FIGURE 1:**
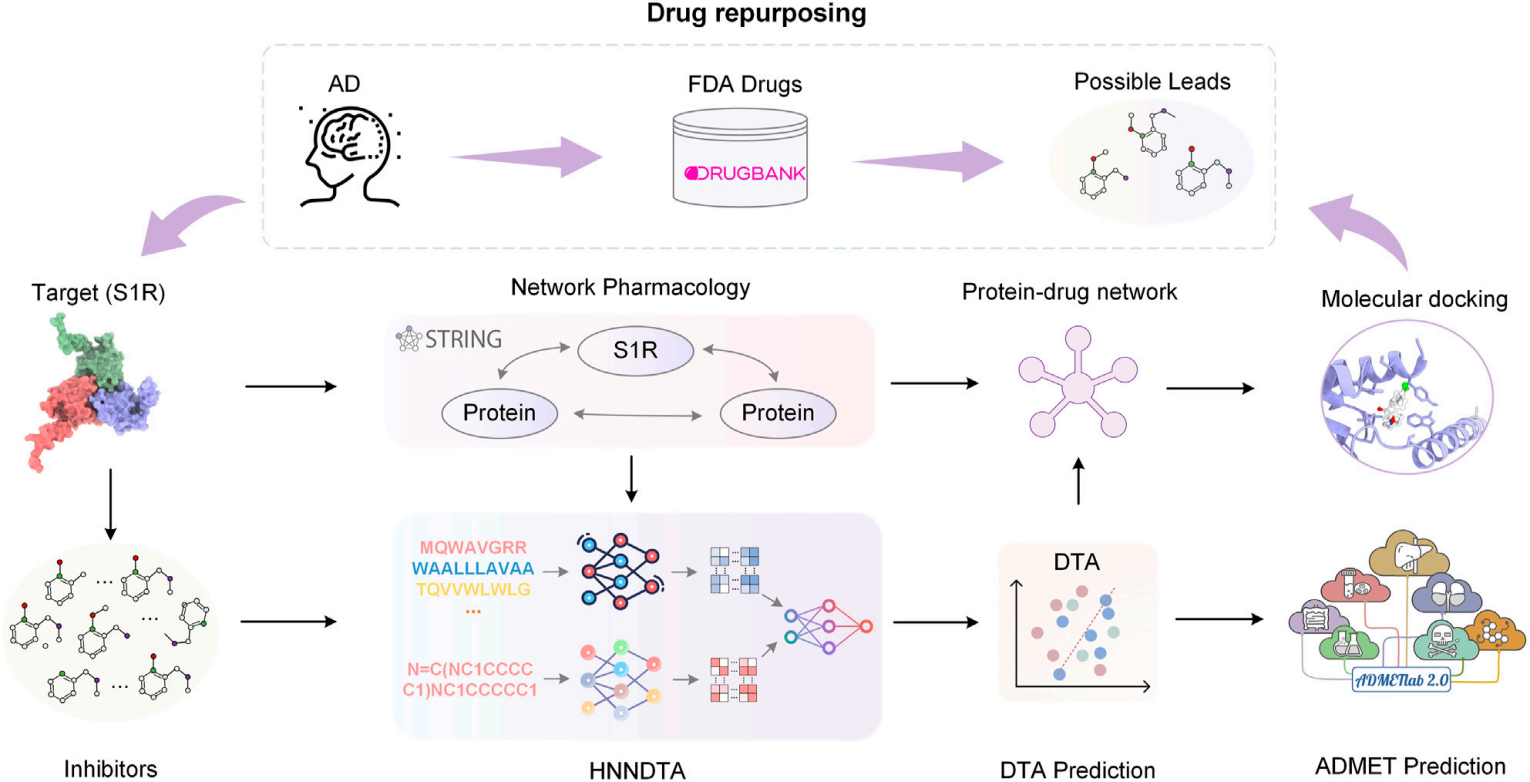
Flowchart of the overall process. In this paper, we started with the AD pathogenic target S1R and conducted PPI analysis to obtain S1R-related proteins. Based on the inhibitors of S1R and related proteins, we constructed a dataset. Subsequently, we trained the dataset using the HNNDTA model. Combining the prediction results of the HNNDTA model and network pharmacology analysis, we screened FDA-approved drugs and obtained candidate drugs. Finally, we performed molecular docking on these candidate drugs, identified lead compounds with potential efficacy, and predicted their ADMET properties.

In this paper, we propose HNNDTA, a hybrid neural network for drug–target affinity prediction, thereby enabling drug repurposing for the treatment of AD. As shown in Figure 1, starting from the pathogenic target of AD, S1R, we conducted protein–protein interaction (PPI) analysis, screened out proteins related to S1R, and constructed a dataset based on inhibitors of S1R and related proteins. Subsequently, we used the HNNDTA model to train the dataset, combined with network pharmacology analysis to screen FDA-approved drugs, and obtained a batch of candidate drugs. Then, we use the molecular docking of candidate drugs with S1R and its related proteins to find potential effective lead compounds, and predict their pharmacokinetics and toxicity to ensure the pharmacokinetics of these candidate drugs. The academic characteristics meet the requirements. Through this series of studies, we have obtained some lead compounds with potential therapeutic effects, which provide new ideas and options for the treatment of AD.

## 2 Materials and methods

### 2.1 Dataset

#### 2.1.1 Target

**FIGURE 2:**
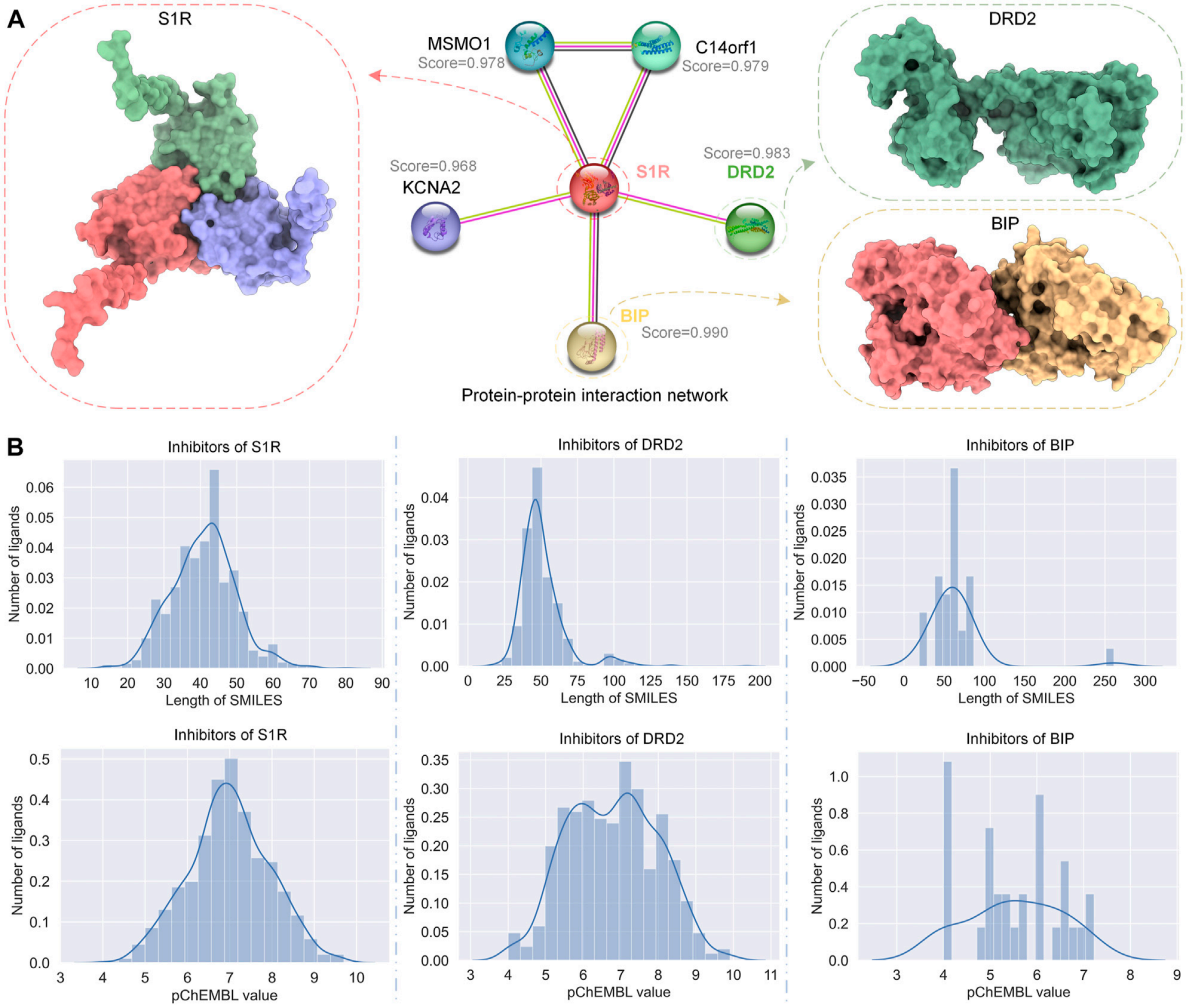
PPI network of S1R and related proteins, DRD2 and BIP, and the distribution of their inhibitor datasets. **(A)** The PPI network of S1R was obtained using STRING, and the 3D structures of the most related proteins BIP and DRD2. **(B)** Distribution of SMILES string lengths and DTA values of the inhibitor datasets for S1R, DRD2, and BIP.

STRING (Szklarczyk et al., 2023) is a database of known and predicted PPIs. We used STRING to get the PPI network of S1R, as shown in Figure 2A; we marked the correlation scores of proteins related to S1R in the network, among which the scores of dopamine D2 receptor (DRD2) and binding-immunoglobulin protein (BIP) are highest, 0.983 and 0.990, respectively, so we picked them as primary targets for network pharmacology analysis. We obtained the sequences of S1R (Q99720), DRD2 (P14416), and BIP (P11021) from the UniProt repository (Consortium, 2019). In addition, we obtained S1R (PDB ID: 5HK1) (Schmidt et al., 2016), DRD2 (6 PDB ID: LUQ) (Fan et al., 2020), and BIP (PDB ID: 3LDN) (Macias et al., 2011) from the RCSB Protein Data Bank (PDB) (Berman et al., 2000), which are 2.51 Å, 3.10 Å, and 2.20 Å, respectively, and their structures are shown in Figure 2A.

#### 2.1.2 Inhibitors

The half-inhibitory concentration (IC_50_) refers to the concentration of the drug or inhibitor required to inhibit half of the specified biological process, and the inhibition constant Ki reflects the inhibitory strength of the inhibitor on the target. The smaller the value, the stronger the inhibitory ability. pIC_50_ is the negative logarithm of the IC_50_ value, which is usually used to characterize the activity of molecules in drug screening. The formula for converting IC_50_ values to pIC_50_ values is

$$pIC50=-\log_{10}\left(IC50\right).$$ (1)

We obtained data on inhibitors of S1R, DRD2, and BIP and their binding abilities to their targets from the ChEMBL database (Gaulton et al., 2012). Although both IC_50_ and Ki can reflect the activity of the inhibitor, for data consistency, we screened the inhibitor data with IC_50_ as the subsequent drug–target affinity (DTA) training data on the HNN. Similarly, under the premise of ensuring the number of datasets, we screened the data whose source description was scientific literature and excluded other data. Figure 2B shows the simplified molecular input line entry system (SMILES) length distribution and binding force distribution of the three protein inhibitors. The inhibitor distribution of S1R and DRD2 showed a Gaussian distribution trend, while the inhibitor distribution of BIP was relatively sparse.

#### 2.1.3 Molecules for drug repurposing

The drug screening library used in this study comes from FDA-approved drugs in the DrugBank database (Wishart et al., 2008). DrugBank is a comprehensive pharmaceutical knowledge bank that provides pharmacists, pharmacologists, health professionals, and drug researchers with free academic resources to help advance drug development and clinical practice. We chose DrugBank as the screening bank because it contains extensive drug information and a list of FDA-approved drugs, which can be used to screen potential drugs for the treatment of AD. These drugs have been proven to be safe and effective treatments in human clinical trials, so they are expected to be used in the treatment of AD. We selected FDA-approved drugs in the DrugBank database as screening libraries, and a total of 2509 drug molecules were available for drug repurposing studies.

### 2.2 HNNDTA

#### 2.2.1 Overview of the framework

**FIGURE 3:**
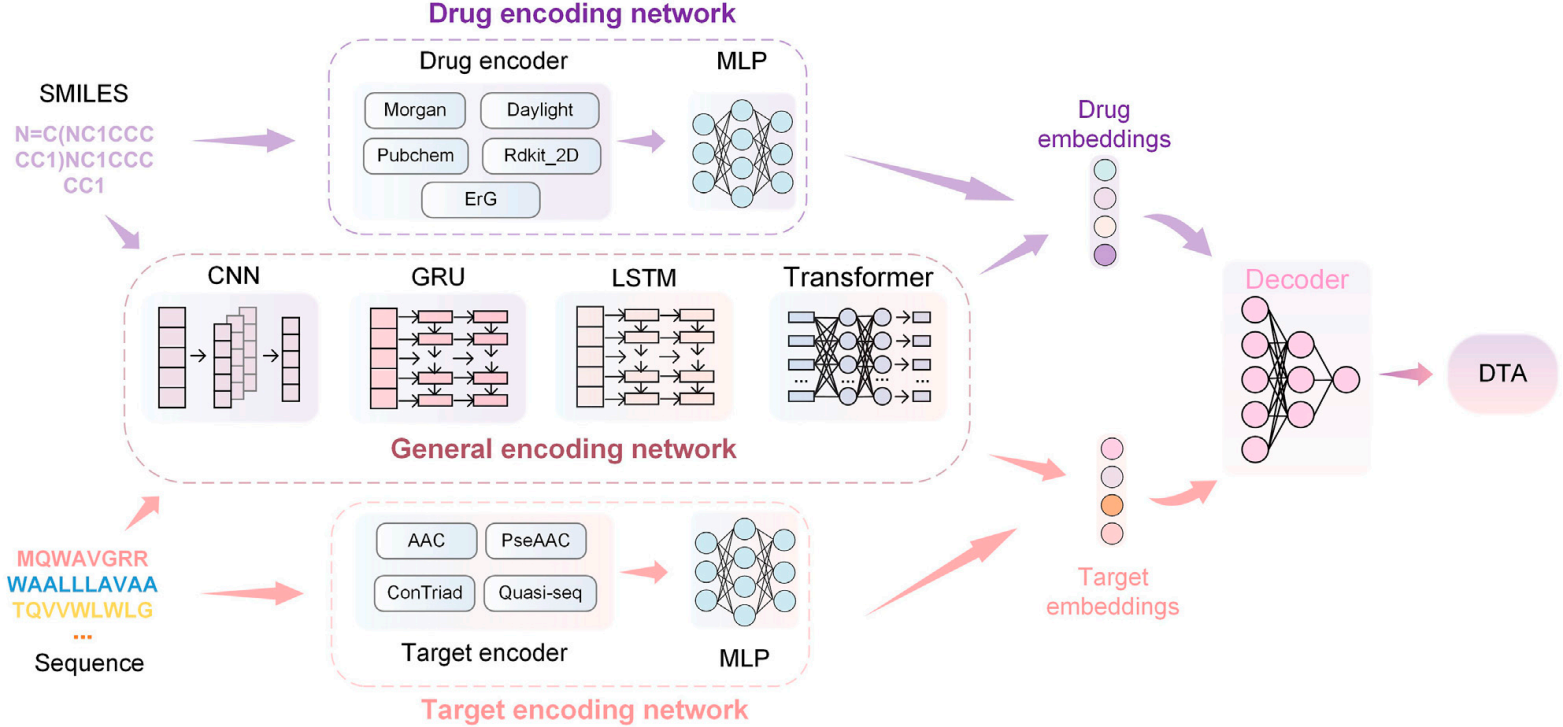
HNNDTA network framework. The framework consists of drug encoding, target encoding, general encoding, and decoding networks. The encoding networks are used to encode the SMILES of drugs and the sequences of proteins to obtain corresponding embeddings. Then, the embeddings are decoded through DNNs to obtain the prediction results of DTA.

The overview of the HNNDTA framework proposed in this study is shown in Figure 3. First, we used a network pharmacology approach to find other targets in the same pathway as the AD target S1R, namely, DRD2 and BIP. We searched the ChEMBL website for inhibitor data for these three targets. The target protein is encoded as a one-dimensional target embedding, and the drug molecule is encoded as a one-dimensional drug embedding. The two encoding vectors are spliced in zero dimension, and after the calculation of the deep neural network (DNN), the final DTA is obtained, which can be expressed as follows:

$$DTA=DNN\left[cat\left(v_{p},v_{d}\right)\right],$$ (2)

where the function $cat\left(a,b\right)$ represents the splicing operation of the 1D a and b vectors, and *v*_*p*_ and *v*_*d*_ represent the encoding vectors of the target protein and the drug molecule, respectively. In this paper, there are 13 kinds of target encoders and 9 kinds of drug encoders, all of which are built by DeepPurpose (Huang et al., 2020). A suitable combined model will produce better prediction accuracy. During the training phase, the dataset was randomly divided into independent training, validation, and test sets in a ratio of 7:1:2. The training set was used to train the model, while the validation and test sets were used to evaluate its performance. Due to the nature of our HNNDTA framework, which was trained on datasets specific to individual targets, it exhibits higher predictive accuracy for single targets. We have observed that models trained on single targets exhibit higher accuracy than those trained on mixed-target datasets.

#### 2.2.2 Drug encoding network

The drug encoder receives SMILES sequences as input. The Morgan encoder first uses the ECFP (Rogers and Hahn, 2010) algorithm to generate the feature representation sequence of the circular substructure of the drug, with a length of 1,024 bits. A multi-layer perceptron (MLP) then processes the sequence of feature representations to obtain a vector representation that can be fed into a neural network. The Morgan encoder is expressed as follows:

$$f_{\mathrm{morg}}\left(SMILES\right)=MLP\left(ECFP\left(SMILES\right)\right).$$ (3)

Similar to the Morgan encoder, the daylight encoder also uses the ECFP algorithm to generate a feature sequence based on the channel substructure of the drug, which is used as the input of the multi-layer perceptron to generate a feature sequence with a length of 2048 bits. The daylight encoder is represented as follows:

$$f_{\mathrm{day}}\left(SMILES\right)=MLP\left(ECFP\left(SMILES\right)\right).$$ (4)

The PubChem encoder (Kim et al., 2019) generates feature sequences using handcrafted important substructures and then generates a feature sequence with a length of 881 bits through a multi-layer perceptron. The PubChem encoder is represented as follows:

$$f_{\mathrm{pub}}\left(SMILES\right)=MLP\left(Substructure\left(SMILES\right)\right).$$ (5)

The rdkit_2d_normalize encoder (Reczko and Bohr, 1994) generates a feature sequence with a length of 200 bits according to the global pharmacophore of the drug and then normalizes the feature sequence by fitting the cumulative density function of a given molecule sample. The rdkit_2d_normalize encoder is represented as follows:

$$\begin{array}{c} f_{\mathrm{rdkit}}\left(SMILES\right)=MLP\left(Normalize\left(Feature\right)\right)\\ Feature=GlobalPharmacophore\left(SMILES\right). \end{array}$$ (6)

The extended reeb graph (ErG) method (Stiefl et al., 2006) mixes the simplified graph and the binding attribute pair to generate a feature sequence and uses the node description of the drug carrier type to encode the relevant molecular properties; the encoded features are obtained after the MLP calculation vector. The ErG coder is expressed as follows:

$$\begin{array}{c} f_{\mathrm{erg}}\left(SMILES\right)=MLP\left(Graph\left(Feature\right)\right)\\ Feature=Scaffold-BasedNodeDescriptor\left(SMILES\right). \end{array}$$ (7)

MLP obtains the output value through feedforward propagation and updates the model parameters through reverse transmission so that the model output value gradually approaches the real value. The output of the MLP forward propagation is expressed as follows:

$$y=AC\left(\overset{M_{l}}{\underset{i=1}{\sum }}\omega_{i,l}•AC\left(\overset{M_{l-1}}{\underset{i=1}{\sum }}\omega_{i,l-1}•\left(\ldots \right)\right)\right),$$ (8)

where *AC* is the activation function and the typical activation function is the modified linear unit ReLU; *M*_*l*_ is the number of neurons in the *l*th layer network, *ω*_*i*,*l*_ is the weight of the *i*th neuron in the *l*th layer network; and the termination condition of $\left(\ldots \right)$ in the aforementioned formula is the first layer of the neural network, that is, the input layer. The reverse transfer uses the Adam optimizer to update the model weights. The underlying algorithm is the gradient descent method. The update on the weight of *ω*_*i*,*l*_ can be expressed as follows:

$$\omega_{i,l}\leftarrow \omega_{i,l}-\eta \frac{\partial E}{\partial \omega_{i,l}},$$ (9)

where *E* is the difference between the predicted value and the real value, $\frac{\partial E}{\partial \omega_{i,l}}$ is the partial derivative of *E* to *ω*_*i*,*l*_, and *η* is the learning rate.

#### 2.2.3 Target encoding network

The input to a target encoder is the amino acid sequence of the target. The signature sequence generated by the amino acid composition (AAC) coder is 8420 positions in length, where each position is consistent with the maximum length of overlapping subsequences (k-mers) of one amino acid. The amino acid composition coder is expressed as follows:

$$AAC_{i}=\frac{f_{i}}{L},\;i=1,2,\ldots ,20,$$ (10)

where *f*_*i*_ represents the number of occurrences of amino acid *i* in the protein and *L* represents the length of the amino acid sequence. The AAC encoder concatenates 20 AAC values for each position in the amino acid sequence to obtain a signature sequence of 8420 elements in length.

The pseudo amino acid composition (PseAAC) encoder adds the hydrophobic and hydrophilic pattern information on the protein based on AAC to generate a 30-bit feature vector representation. The pseudo-amino acid composition encoder is expressed as follows:

$$\begin{array}{c} PseAAC_{i,j}=\frac{\sum_{k=1}^{L}f_{k,i}w_{k,j}}{\sum_{k=1}^{L}f_{k,i}},\\ \;i=1,2,\ldots ,20;\;j=1,2,\ldots ,30, \end{array}$$ (11)

where *f*_*k*,*i*_ represents the frequency of amino acid *i* in the *k*th position in the protein sequence and *w*_*k*,*j*_ represents the weight of the pattern of the *k*th amino acid and the relative position *j*. The PseAAC encoder concatenates 30 PseAAC values for each position in the amino acid sequence, resulting in a feature vector of 30 elements in length.

The conjoint triad (ConTriad) encoder (Shen et al., 2007) forms a 7-letter alphabet based on amino acid triplet features, generating a feature vector with a length of 343 elements. The ConTriad encoder is expressed as follows:

$$ConTriad_{i}=\frac{\sum_{j=1}^{7}f_{j,i}w_{j}}{L-2},\;i=1,2,\ldots ,3430,$$ (12)

where *f*_*j*,*i*_ indicates that the three adjacent amino acids in the protein sequence are converted into a number according to the 7-letter alphabet, the *i*th element indicates the frequency of the *j*th triplet appearing in the protein sequence, and *w*_*j*_ is the weight of the *j*th triplet. The ConTriad encoder concatenates 343 ConTriad values for each position in the amino acid sequence, resulting in a feature vector of 343 elements in length.

The quasi-sequential encoder consists of a 100-element feature vector of quasi-sequential descriptors (Chou, 2000). The feature vectors generated by the aforementioned manual feature encoder will be further processed as input to MLP to obtain the feature vector of the target. The quasi-sequential encoder is expressed as follows:

$$QuasiSeq_{i}=\;\sum \frac{{\left(j=1\right)}^{N}\rho_{j}}{d_{ij}},\;i\;=\;1,\;2,\;\ldots ,\;100,$$ (13)

where *ρ*_*j*_ represents the weight of the *j*th quasi-sequential descriptor and *d*_*ij*_ is the distance between the *i*th amino acid and the *j*th sequence descriptor. The quasi-sequential encoder concatenates 100 QuasiSeq values for each position in the amino acid sequence, resulting in a feature vector of 100 elements in length.

#### 2.2.4 General encoding network

The aforementioned drug and target feature extraction methods are based on prior chemical knowledge and manual transformation, so these encoders cannot be mixed. The encoders introduced in this section are general-purpose encoders based on DNNs, including convolutional neural networks (CNNs) (Krizhevsky et al., 2017), gated recurrent units (GRUs) (Chung et al., 2014), long short-term memory (LSTM) (Hochreiter and Schmidhuber, 1997), and transformers (Vaswani et al., 2017). These neural networks treat amino acid sequences as one-dimensional data.

The CNN encoder is a multilayer 1D CNN (Krizhevsky et al., 2017). After encoding the amino acid sequence character by character, the obtained deep feature vector will pass through multiple 1D convolutional layers and finally pass through the one-dimensional maximum pooling layer to obtain the output of the target feature vector. The output of the 1D convolutional layer is the result of convolving the input with the convolution kernel, which can be expressed as follows:

out=input⊗kernel, (14)

where *⊗* represents a convolution operation. Assuming that the convolution kernel size is 2*k* + 1, *k* ∈ *N* ^+^, the *i*th convolution output can be expressed as follows:

$$out_{i}=\overset{i+k}{\underset{j=i-k}{\sum }}\overset{2k+1}{\underset{a=1}{\sum }}input_{j}•kernel_{a}.$$ (15)

GRU and LSTM encoders are types of recurrent neural networks. In both networks, each node will get an output based on the state at the last moment and the current input and update the state of the node. This can solve the problem of traditional convolutional networks without long-term memory to a certain extent. Specifically, the SMILES sequence or amino acid sequence will first pass through the CNN for feature extraction and then use the output of the CNN as the input of the recurrent network.

The transformer encoder applies a self-attention mechanism (Vaswani et al., 2017). Due to the computational time and memory cost of the transformer, amino acid sequences are decomposed into moderately sized protein substructures, such as motifs, and each segmentation is then treated as a token and fed into a self-attention-based encoder. If a SMILES sequence or amino acid sequence is treated as a sentence, cut into several meaningful phrases, and encoded into several vectors with the same number of phrases, denoted as x, then the output of the transformer can be expressed as

$$\begin{array}{c} x_{1}\;=\;\mathrm{norm}\left(x+attn\left(x,\;mask\right)\right),\\ out=\mathrm{norm}\left(x_{1}+feedforward\left(x_{1}\right)\right), \end{array}$$ (16)

where *attn* is a self-attention function, *mask* is a Boolean value about whether the input *x* is eliminated, feedforward is a feedforward neural network, and norm is a layer normalization operation.

#### 2.2.5 Evaluation metrics

In mathematical statistics, mean-squared error (MSE) is a method used to measure the difference between the predicted and real values. It calculates the mean of the squared difference between predicted and true values, which is the expected value of the squared difference between predicted and true values. The smaller the value of the MSE, the higher the prediction accuracy of the prediction model. Assuming there are n samples, MSE can be expressed by the following formula:

$$MSE=\frac{1}{n}\overset{n}{\underset{i=1}{\sum }}{\left(y_{i}-{\hat{y}}_{i}\right)}^{2},$$ (17)

where *y*_*i*_ and $\hat{y}i$ are the true and estimated values of the *i*th sample, respectively. In this paper, the MSE is used to evaluate the accuracy of the model to predict the binding affinity of the drug to the target.

Harrell’s C-index (also known as the concordance index, CI) is a widely used metric for evaluating the performance of risk models. It is commonly employed in survival analysis, especially when dealing with censored data (Harrell et al., 1982). The C-index measures the degree of concordance between predicted and observed rankings of survival times. It serves as an indicator of the model’s accuracy with values closer to 1, indicating a higher level of consistency between the predicted outcomes and the actual observed outcomes.

Suppose the data are represented by vectors $\left(\tilde{T_{i}},\Delta_{i},X_{i1},\ldots ,X_{ip}\right),\;i=1,\ldots ,n$ , where $\tilde{T_{i}}$ is a possibly right-censored continuous survival time and ${\left(X_{i1},\ldots ,X_{ip}\right)}^{T}$ is a vector of predictor variables. It is assumed that $\tilde{T_{i}}$ is the minimum of the true survival time *T*_*i*_ and an independent continuous censoring time *C*_*i*_. The variable Δ_*i*_≔*I*(*T*_*i*_ < = *C*_*i*_) indicates whether *T*_*i*_ has been fully observed (Δ_*i*_ = 1) or not (Δ_*i*_ = 0). A one-dimensional score $\eta_{i}\in R$ is estimated for each observation *i* = 1, …, *n*, by averaging the cumulative hazard estimates over all trees and all time points. The concordance index is given by

$$C=\frac{\sum_{i,j}I\left(\tilde{T_{i}}>\tilde{T_{j}}\right)\cdot I\left(\eta_{j}>\eta_{i}\right)\cdot \Delta_{j}}{\sum_{i,j}I\left(\tilde{T_{i}}>\tilde{T_{j}}\right)\cdot \Delta_{j}},$$ (18)

where the indices *i* and *j* refer to pairs of observations in the sample (Schmid et al., 2016).

### 2.3 Network pharmacology

Network pharmacology (NP) (Hopkins, 2008) is a new drug development method based on systems biology. It reveals the multi-target action mechanism of drugs by integrating protein interaction and drug compound networks. To construct a network pharmacology-based analysis, we mapped protein–protein and protein–drug interaction networks (Hasan et al., 2020). We fetch the PPI network from the STRING database and select the protein most related to the target we need to study. We then used the HNNDTA model to predict the binding forces between these proteins and compounds. To identify the best compounds, we picked the top 20 most binding proteins for each protein and mapped them into a protein–compound network. We use Sankey diagrams (Lee et al., 2019) to visualize drug–protein interaction networks to better understand and analyze the mechanism of action of drugs in biological systems. In this network, we can identify which compounds may be the most promising drug candidates by analyzing the interactions between proteins and compounds. In particular, for those compounds that bind strongly to multiple proteins, we can select them as our drug candidates.

### 2.4 Molecular docking

In molecular docking tasks, AutoDock Vina (Eberhardt et al., 2021) is one of the widely used docking engines in AutoDock Suite, and its open-source code and fast docking speed are favored. We use AutoDock Vina 1.1.2 for molecular docking experiments. First, we obtained the 3D molecular structure files of all receptor molecules and processed them to remove crystal water and hydrogenate them to generate preprocessed receptors. Next, we first removed the crystal water and the original ligand, then added hydrogen and charge distribution, and manually set the active site area of the receptor as the grid box according to the feature information on the protein in UniProt. For the ligand file, we obtained its structure from PubChem (Kim et al., 2019) and then performed hydrogenation and charge addition to obtain the preprocessed ligand file. Then, we used AutoDock Vina for docking; exhaustiveness is set to 32; a total of 10 docking poses are generated; the top 5 best poses are kept, and finally, the binding energy value (in k/mol) of the best pose is used as the docking score. The results of molecular docking were output in pdbqt format and visualized and analyzed using PyMOL molecular visualization software. The docking results are evaluated by factors such as hydrogen bonds, van der Waals forces, and electron static energy.

## 3 Results

### 3.1 Performance evaluation

**FIGURE 4:**
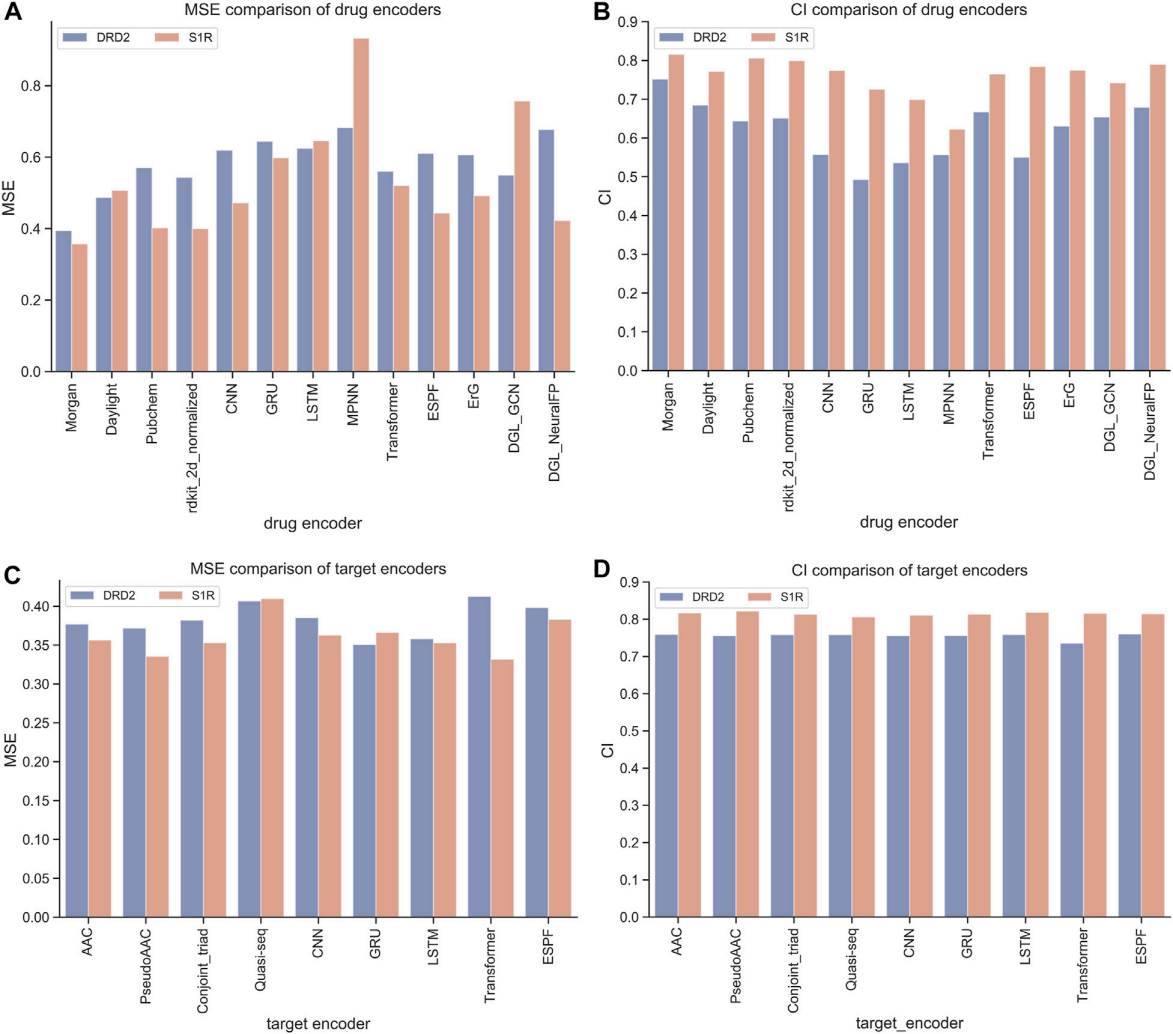
Performance comparison of drugs and encoders. **(A, B)** Comparison of MSE and CI of drug encoders. **(C, D)** Comparison of MSE and CI of target encoders.

The HNNDTA framework was constructed using 13 drug encoders and 9 target encoders. We fixed the target encoder as AAC and constructed 13 different drug encoder models. The MSE and CI of the test models are shown in Figures 4A, B. The orange column is the test result of the ligand dataset of the S1R protein, and the sky blue column is the test result of the ligand dataset of the DRD2 protein. The smaller the MSE and the larger the CI, the smaller the difference between the predicted results of the model and the real results, and the higher the accuracy of the model. In the figure, the MSE value of the Morgan encoder is the smallest and the CI value is the largest, indicating that the Morgan encoder will make the model perform better, and the Morgan encoder should be considered in the subsequent grid search for the best drug encoder–target encoder combination. We fixed the drug encoder as Morgan, constructed nine models with different target encoders, and compared the MSE and CI of the test models, as shown in Figures 4C, D. The MSE values of each encoder are basically at the same level because there is only a very small amount of target data in the dataset, and the difference in information provided by the target is less.

**FIGURE 5:**
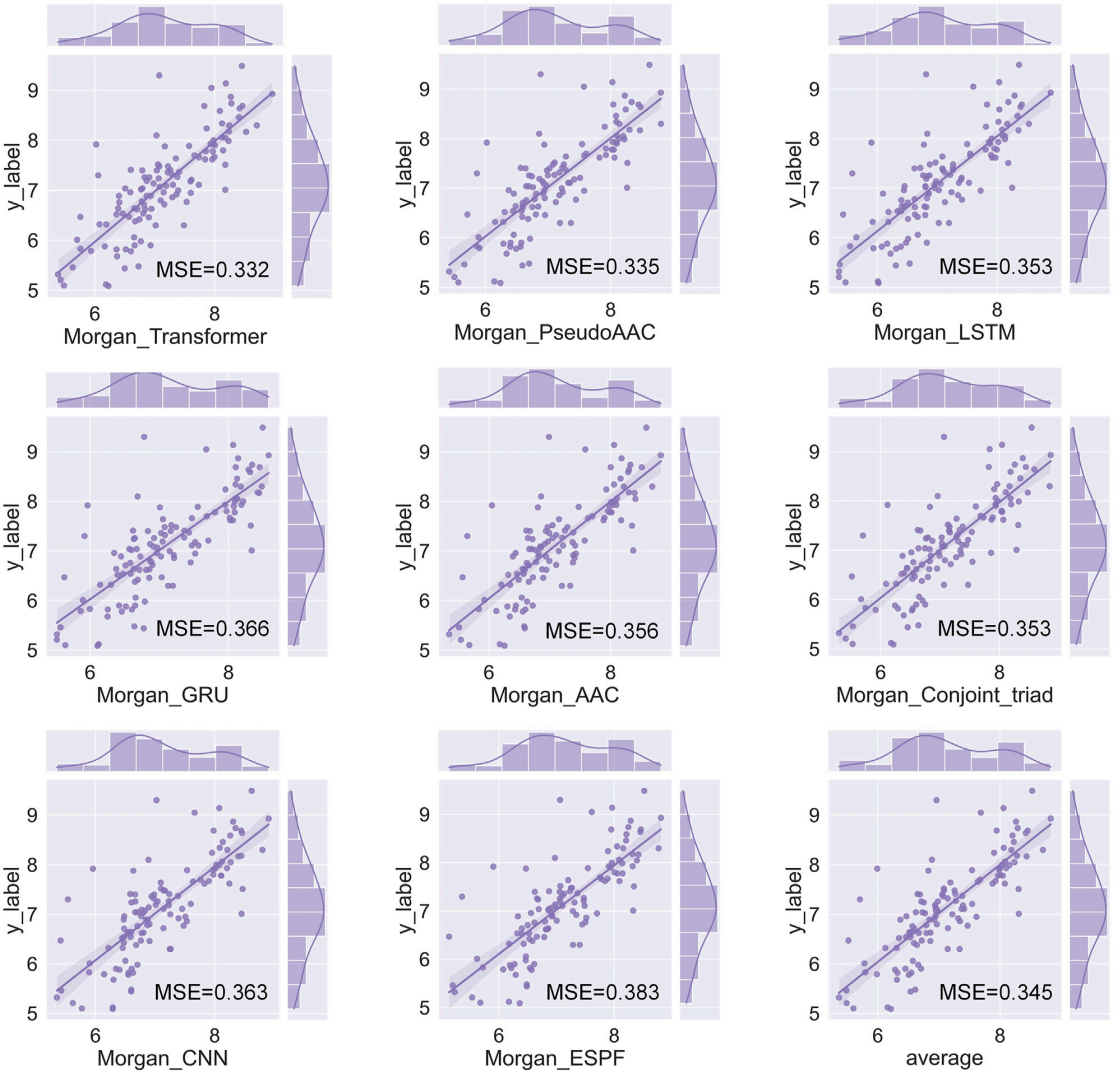
Fit plot of the best-performing model.

We have a total of 117 models of 13 drug encoders and 9 target encoders, and conduct a grid search on the ligand datasets of the three targets of S1R, DRD2, and BIP to find the best models. After testing, there are eight models with both MSE and CI in the top 10, as shown in Figure 5; Table 1. Among them, the Morgan encoder has the best encoding effect on drugs, and the transformer and PseudoAAC encoders have better encoding effects on protein targets. Overall, the performance of these eight models is comparable and complements each other. In the next step of screening candidate drugs, the average of the votes predicted by the eight models is taken as the drug–target interaction score.

**TABLE 1:** Best-performing model.

Drug encoder	Target encoder	MSE	CI
Morgan	Transformer	0.332	0.816
Morgan	PseudoAAC	0.335	0.822
Morgan	LSTM	0.353	0.818
Morgan	Conjoint_triad	0.353	0.813
Morgan	AAC	0.356	0.817
Morgan	CNN	0.363	0.811
Morgan	GRU	0.366	0.814
Morgan	ESPF	0.383	0.815

### 3.2 Virtual screening of HNNDTA and network pharmacology

In this study, 2506 FDA-approved drugs were used as drug candidates for the AD target protein S1R and other targets of the same pathway, DRD2 and BIP. For S1R and DRD2, the respective models were trained using ligand datasets obtained from the ChEMBL website. For BIP, due to the lack of ligand data on BIP on the ChEMBL website, it is not enough to train a good model. We can pre-train the model with a large amount of ligand data for the same pathway target of S1R and then fine-tune the model with the ligand data on BIP itself.

**FIGURE 6:**
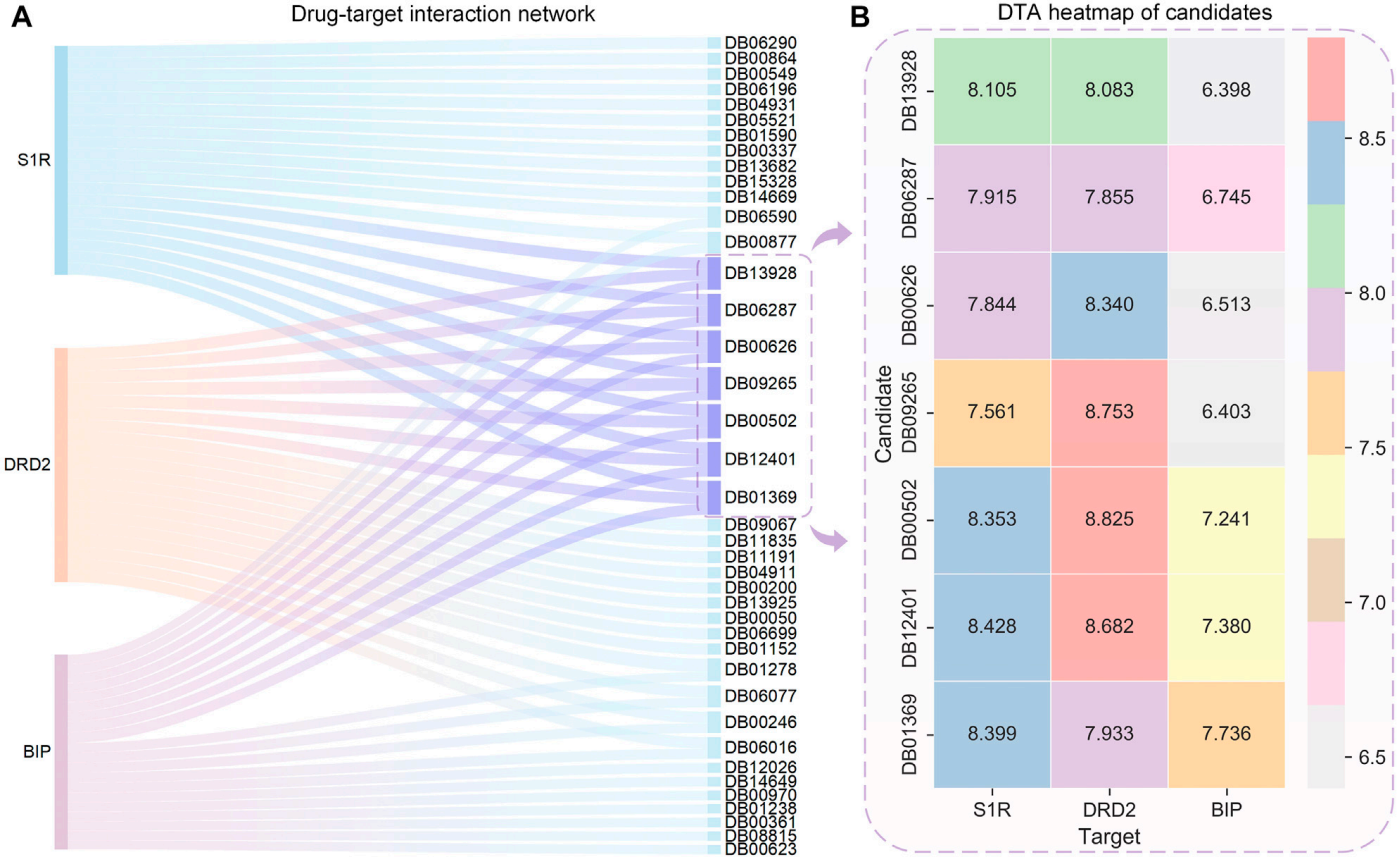
Sankey diagram of the DTI network for the top 20 drugs with the highest affinity to S1R, DRD2, BIP, and DTA heatmaps of the candidates. **(A)** Sankey diagram of the drug–target interaction network is shown, with the sky-blue nodes indicating the selected candidates. **(B)** Binding affinity heatmap of the candidates with S1R, DRD2, and BIP. Red represents the highest DTA value, while gray represents the lowest DTA value. Both DB12041 and DB00502 exhibit high affinity to S1R and DRD2.

The HNNDTA model was used to predict the activities of FDA-approved drugs and targets S1R, DRD2, and BIP, and the 20 drugs with the highest binding activities to these three targets are shown in Figure 6A. On the left side of the Sankey diagram are the three target proteins, and on the right side are the 20 drugs with the highest binding activity to these three targets. At the intersection, there are a total of 40 drugs. The prediction results show that most drugs can only have high activity with one or two targets, while the seven drugs DB13928, DB06287, DB00626, DB09265, DB00502, DB12401, and DB01369 have high binding activity with three targets, indicating that they can simultaneously inhibit these three AD-related targets. Therefore, these seven drugs can be used as alternative drugs for the treatment of AD. The DTA values of the aforementioned seven candidate drugs and S1R, DRD2, and BIP are shown in Figure 6B. The DTA values of these seven drugs and three targets stand out among more than 2,000 FDA drugs. The two drugs, DB00502 and DB12401, have the highest combined affinity for the three targets and are expected to become candidate drugs for the treatment of AD.

### 3.3 Benchmark testing

To assess the accuracy of the model predictions and validate the efficacy of the drugs identified through network pharmacology (i.e., haloperidol and bromperidol), benchmark testing was conducted. Known high-affinity ligands for S1R, DRD2, and BIP were collected from the ChEMBL and BindingDB databases for validating the docked scores of the screened drugs as being higher than or comparable to the known high-affinity ligands. Conversely, known low-affinity ligands were gathered to demonstrate that the docked scores of the screened drugs are superior to them. The information on known ligands and their affinities is presented in Table 2.

**TABLE 2:** Collected known drug–target pairs with high and low binding affinities.

Target	Inhibitor	Affinity	Type
S1R	Haloperidol	8.54	Ki
S1R	Donepezil	7.84	Ki
S1R	Fluvoxamine	7.44	Ki
S1R	Corticosterone	4.45	Ki
S1R	Cocaine	5.05	Ki
DRD2	Haloperidol	8.76	Ki
DRD2	Pimozide	7.93	Ki
DRD2	Amisulpride	7.90	Ki
DRD2	Procaterol	4.07	Ki
DRD2	Isoproterenol	4.32	Ki
BIP	CHEMBL462871	7.22	Kd
BIP	CHEMBL516197	4.85	Kd

First, the HNNDTA model was utilized to predict the binding affinities (pIC50) of the collected ligands to the three targets. The prediction results are shown in Table 3, where the green boxes and red boxes represent known high- and low-affinity drug–target pairs, respectively. Overall, the predicted affinities in the green boxes are higher than those in the red boxes, indicating that our model can accurately differentiate between high and low affinities among drug–target pairs. Subsequently, blind docking of ligand–protein was performed using QuickVina-W software (Hassan et al., 2017), and the docking scores are presented in Table 4. Lower docking scores indicate smaller binding energies and higher binding affinity. The docking scores in the green boxes are generally lower than those in the red boxes, suggesting the effectiveness of the docking procedure.

**TABLE 3:** DTA predicting results obtained from HNNDTA are presented. The green boxes and red boxes represent known high-affinity and low-affinity drug–target pairs, respectively.

Ligand targets	DB00502	DB12401	DB00843	DB00176	DB04652	DB00907	DB01100	DB06288	DB01366	DB01064	CHEMBL462871	CHEMBL516197	DB15477
S1R	7.5	7.3	6.8	6.3	6.7	6.5	6.9	6.0	6.3	6.3	6.3	5.6	6.8
DRD2	7.8	7.9	5.9	5.7	5.7	5.4	6.4	8.5	5.4	5.2	5.9	5.2	5.9
BIP	6.8	6.7	5.3	5.0	5.4	5.0	5.4	7.3	4.4	4.5	6.3	4.4	5.4

**TABLE 4:** The molecular docking results obtained from QuickVina-W are presented, where a lower docking score indicates weaker binding energy and stronger binding affinity. The green boxes and red boxes represent known high-affinity and low-affinity drug–target pairs, respectively.

Ligand targets	DB00502	DB12401	DB00843	DB00176	DB04652	DB00907	DB01100	DB06288	DB01366	DB01064	CHEMBL462871	CHEMBL516197	DB15477
S1R	−10.3	−10.3	−10.5	−8.3	−6.5	−8.7	−11.3	−5.4	−5.7	−6.8	−8.1	−7.5	−6.7
DRD2	−11.3	−10.7	−9.5	−7.5	−8.3	−7.3	−10.9	−7.9	−7.0	−7.2	−10.7	−6.9	−8.5
BIP	−8.4	−8.6	−8.4	−7.3	−7.4	−7.7	−10.1	−7.6	−6.6	−6.9	−9.2	−7.6	−8.3

Our screened drugs, haloperidol and bromperidol, exhibit lower overall docking scores with the three targets compared to most other drugs. Furthermore, the docking scores of the screened drugs are comparable to those of known high-affinity ligands and significantly lower than those of known low-affinity ligands. This indicates that the HNNDTA model successfully identified high-affinity drugs suitable for multiple targets. It is worth noting that Table 4 shows that the drug pimozide has the best multi-target docking score. However, molecular docking requires manual preprocessing of 3D structures and is computationally time-consuming, making it difficult to apply to high-throughput drug target screening in network pharmacology. The HNNDTA model can expedite this process and successfully screen multi-target high-affinity drugs, even if it may represent a suboptimal solution.

### 3.4 Virtual screening of molecular docking

Small molecules have smaller molecular weights, which favor better pharmacokinetics and less toxicity. The molecular weight of antibiotics is large, and the metabolic process affects the drug’s efficacy. Small molecules have good medicinal properties, such as high bioavailability, good tissue specificity, and low toxicity and side effects, and are suitable for drug research and development. Therefore, we only choose small molecules with a weight of less than 500 as lead compounds. In AutoDock Vina docking, we use the binding energy value of the best pose as the docking score and tabulate the results in Table 5. The molecules of DB09265 and DB13928 are very large, beyond the active site region of the receptor, causing errors in Vina, which indicates that the binding between the two ligands and the receptor is difficult. Since Vina uses binding energy as a docking score, a smaller score indicates tighter binding between the two molecules, which generally indicates better docking. However, when the score is positive, it means that docking is difficult to produce. Both of these conditions can indicate a docking failure. Table 5 shows that although the ligands DB01369 and DB06287 have good docking effects on DRD2 and BIP receptors, they are difficult to bind to S1R receptors. Ligands DB00502 (bromperidol) and DB12401 (haloperidol) have good binding abilities to the three receptors, and the molecular weight is less than 500, meeting the screening requirements, so they may become potential drugs for AD.

**TABLE 5:** Overview of candidate compounds and their docking scores with S1R, DRD2, and BIP proteins. The docking scores were calculated using the molecular docking software application AutoDock Vina, with higher scores indicating stronger interactions.

DrugBank ID	Generic name	Summary	Docking score		
			S1R	DRD2	BIP
DB00502	Haloperidol	Antipsychotic	**−8.856**	−7.265	−8.585
DB00626	Bacitracin	Antibiotic	−7.979	−6.412	−6.35
DB01369	Quinupristin	Antibiotic	**-**	**−9.455**	**−9.689**
DB06287	Temsirolimus	Antineoplastic	**-**	**−9.674**	**−9.858**
DB09265	Lixisenatide	GLP-1 receptor agonist	-	-	-
DB12401	Bromperidol	Antipsychotic	**−8.516**	−7.031	−8.245
DB13928	Semaglutide	Peptide 1 receptor agonist	-	-	-

The bold values indicate the docking scores of the top two drugs with the highest docking scores for a specific target.

### 3.5 Explanatory analysis of DTA

**FIGURE 7:**
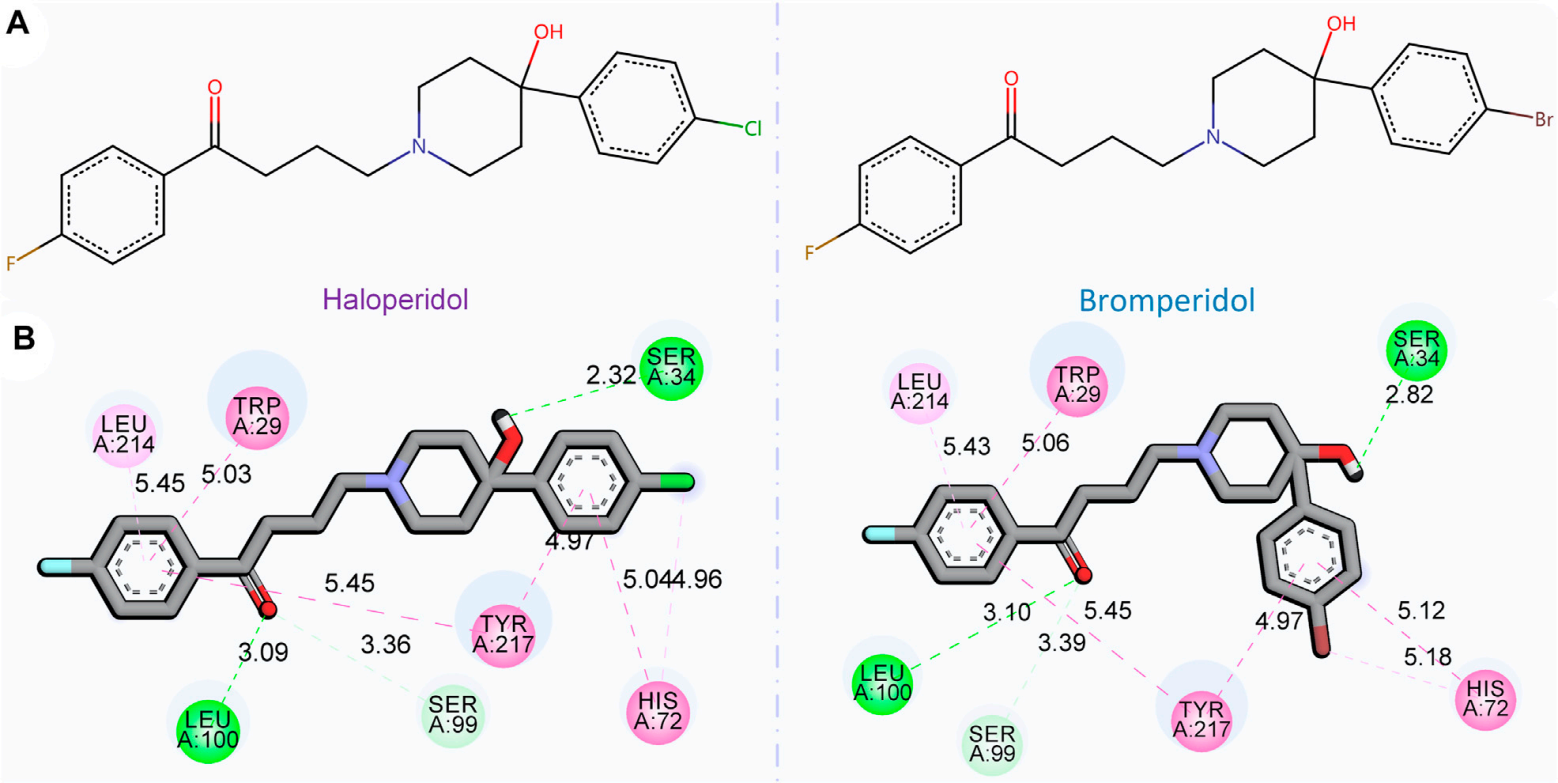
2D chemical structures of haloperidol and bromperidol and their 2D poses generated by docking with the target S1R. In the 2D chemical structures **(A)** and 2D docking poses **(B)**, the chemical structures of haloperidol and bromperidol are shown on the upper part and their 2D poses generated by docking with S1R are shown on the lower part. Although their chemical structures are very similar, their affinities to S1R differ when binding to it. This may be due to the different interaction distances between the halogen atoms in the bromperidol molecule and the six amino acids of S1R.

Figure 7 shows the 2D chemical structures of haloperidol and bromperidol, and the 2D poses resulting from docking with the target S1R. As shown in Figure 7A, their chemical structures are very similar, differing only by one halogen atom: haloperidol with a Cl atom and bromperidol with a Br atom. They are both high-affinity ligands for S1R, with only slight differences. This may be caused by the different interaction distances between the halogen atoms in the bromperidol molecule and the six amino acids of S1R. As shown in Figure 7B, both drugs produced hydrogen bonds with SER34, SER99, and LEU100 amino acids of S1R, and produced *π* − *π* interactions with TRP29, HIS72, LEU214, and TYR217 amino acids of S1R. The two molecules stabilize the association between them through their interaction with S1R.

**FIGURE 8:**
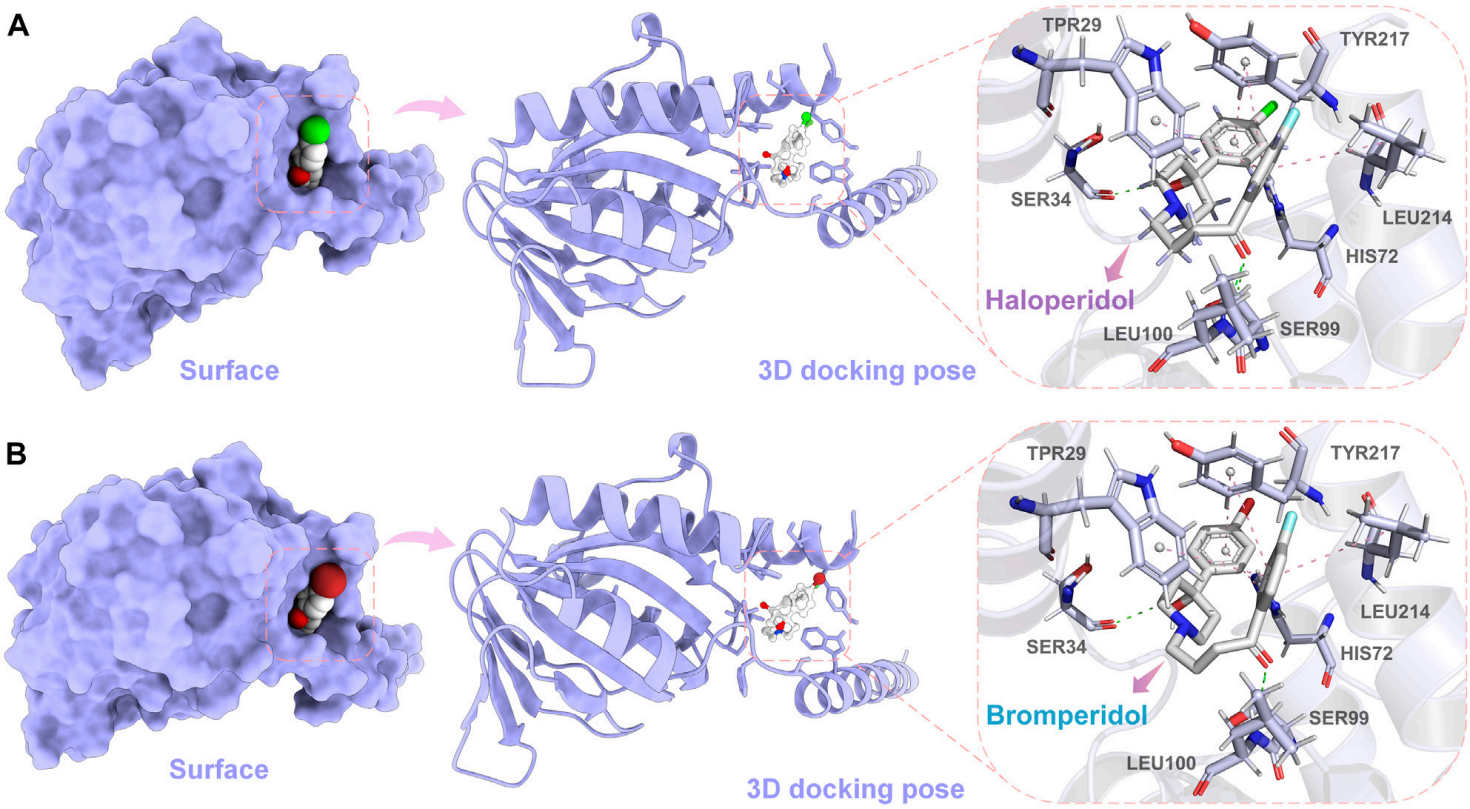
Simulation results of 3D docking of haloperidol and bromperidol with the target S1R. Through docking simulation, we demonstrated the surface and 3D docking poses of haloperidol **(A)** and bromperidol **(B)** with S1R. In the 3D docking poses, green represents hydrogen bonding and pink represents *π* − *π* interactions. The two molecules stabilize their binding through interactions with S1R.

In order to further observe the docking poses of haloperidol and bromperidol with S1R, we also plotted the 3D docking simulation results, as shown in Figure 8. Both haloperidol and bromperidol dock at the S1R surface and interact with surrounding S1R amino acids. As shown in Figures 8A, B, the docking poses of haloperidol and bromperidol are very close to S1R, which is related to their similar chemical structures. They jointly participate in the stable combination with S1R and produce more interactions.

**FIGURE 9:**
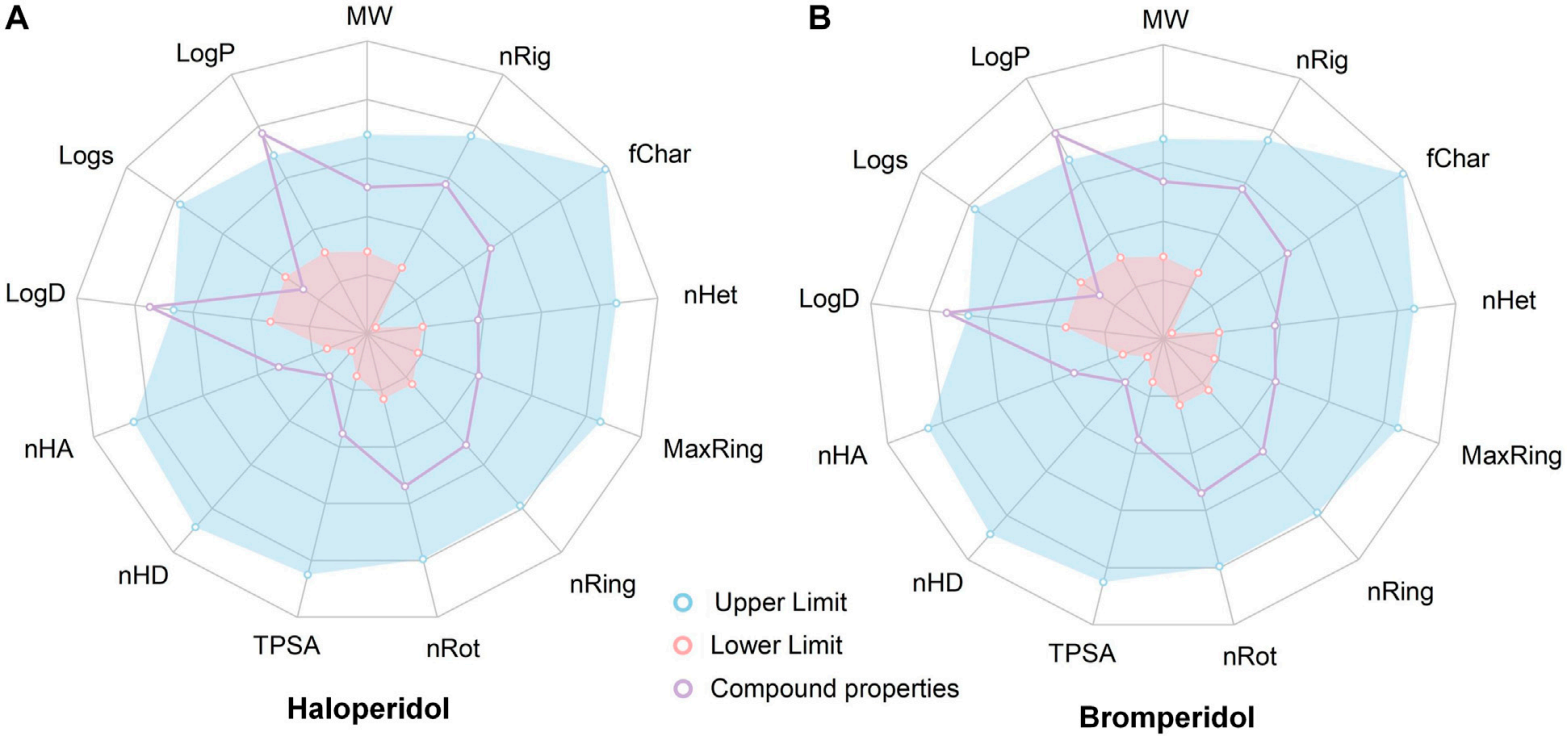
Results of the ADMET evaluation of haloperidol and bromperidol by the ADMETlab 2.0 tool. The evaluation results of haloperidol **(A)** and bromperidol **(B)** are generally similar, with compound properties distributed between the upper and lower limits, except for logD and logP.

To evaluate the ADMET, of haloperidol and bromperidol, we evaluated them using the ADMETlab 2.0 tool (Xiong et al., 2021), as shown in Figure 9. The evaluation results of haloperidol and bromperidol are roughly similar, except for logD and logP, and their compound properties are distributed between the upper and lower limits. This shows that haloperidol and bromperidol have better pharmacokinetic conditions and almost no toxicity. Haloperidol is an antipsychotic drug used to treat schizophrenia and other psychotic disorders, as well as symptoms of agitation, irritability, and delirium. Bromperidol is used to treat schizophrenia and other psychotic symptoms and has been used in trials investigating the treatment of dementia, depression, schizophrenia, anxiety disorders, and psychosomatic disorders, among others. It further illustrates the accuracy of our HNNDTA screening by finding a trial that is already in the treatment of AD and, at the same time, screening a new potential drug for the treatment of AD.

## 4 Discussion

Alzheimer’s disease is a significant age-related illness that has garnered widespread attention in society. In this article, we propose a drug-screening framework that combines network pharmacology and hybrid neural networks to discover potential drugs for treating Alzheimer’s disease. Existing evidence supports S1R as a potential therapeutic target for Alzheimer’s disease. Initially, we conducted protein–protein interaction analysis using the STRING database to identify the most relevant targets associated with S1R, including DRD2 and BIP. These targets were then utilized in network pharmacology for drug screening. We developed a hybrid neural network framework to predict the binding affinity between targets and ligands, enabling the prediction of multi-target interactions for drug candidates. Benchmark testing was performed using a collection of known ligands with high and low affinity, demonstrating our model’s ability to differentiate between high- and low-affinity ligands. Furthermore, our model identified two drugs, haloperidol and bromperidol, with overall higher docking scores than other drugs, thereby validating the effectiveness of our proposed framework.

In PPI analysis, our results indicated that BIP and DRD2 have a higher combined score than other proteins related to S1R. A substantial body of evidence suggests that S1R, in combination with BIP, a regulator of endoplasmic reticulum stress (ERS), plays a pivotal role in the ERS pathway, which is a component of cellular stress and a core mechanism underlying synaptic loss and neurodegeneration in AD pathology (Ortega-Roldan et al., 2013; Venkataraman et al., 2022). S1R-dependent neuroprotection is likely to be mediated by the regulation of the unfolded protein response (UPR) in ERS (Voronin et al., 2023). Under ERS conditions, S1R agonists promote the dissociation of S1R-BIP calcium ion-sensitive chaperone complexes, resulting in enhanced chaperone activity of BIP toward misfolded proteins and S1R binding to client protein IRE1*α*. The regulatory effect of S1R agonists can increase the expression of BIP and brain-derived neurotrophic factor (BDNF) and decrease the expression of pro-inflammatory interleukin-6 (IL-6) (Hayashi and Su, 2007; Rosen et al., 2019; Zhemkov et al., 2021). Thus, S1R agonist regulation presents a viable strategy for the neuroprotective treatment of AD, aimed at reducing ERS and neuroinflammation while enhancing neural plasticity (Voronin et al., 2023).

It should be noted that the HNNDTA model does not differentiate between ligands as agonists or antagonists of the targets. Unfortunately, the existing literature reports that haloperidol is an antagonist of S1R (Maurice and Su, 2009), while S1R agonists are potential drugs for treating AD. Therefore, haloperidol is not suitable for the treatment of AD. On the other hand, bromperidol, which was selected by the HNNDTA model, may be the optimal candidate drug for AD treatment. The existing literature has discussed the potential of antipsychotic drugs, including bromperidol, on multiple targets related to AD (Kumar et al., 2017).

## Data Availability

The original contributions presented in the study are included in the article/Supplementary material, further inquiries can be directed to the corresponding author.
